# Pediatric respiratory pathogen dynamics in Southern Sichuan, China: a retrospective analysis of gender, age, and seasonal trends

**DOI:** 10.3389/fped.2024.1374571

**Published:** 2024-07-17

**Authors:** Qing Li, Min Song, Zhi Hu, Yinhuan Ding, Chengliang Huang, Jinbo Liu

**Affiliations:** ^1^ Department of Laboratory Medicine, The Affiliated Hospital of Southwest Medical University, Luzhou, China; ^2^ Sichuan Province Engineering Technology Research Center of Molecular Diagnosis of Clinical Diseases, The Affiliated Hospital of Southwest Medical University, Luzhou, China; ^3^ Molecular Diagnosis of Clinical Diseases Key Laboratory of Luzhou, The Affiliated Hospital of Southwest Medical University, Luzhou, China; ^4^ Department of Medical Laboratory, Southwest Medical University, Luzhou, China; ^5^ Department of Respiratory and Critical Care Medicine, The Affiliated Hospital of Southwest Medical University, Luzhou, China

**Keywords:** respiratory tract infections, pediatric epidemiology, pathogen prevalence, diagnostic strategies, public health policy, Southern Sichuan

## Abstract

**Objective:**

To address the research gap in the epidemiology of pediatric respiratory tract infections (RTIs) in Luzhou, Southern Sichuan, China, by analyzing respiratory pathogens in a large pediatric cohort from 2018 to 2021, covering the pre- and during-COVID-19 periods.

**Methods:**

This study conducted a retrospective analysis of children with RTIs in Luzhou from July 2018 to January 2021. Strict exclusion criteria were applied to ensure an accurate representation of the pediatric population. Pathogen detection included viruses, bacteria, and atypical agents.

**Results:**

Pathogens were identified in 52.8% of 12,546 cases. Viruses accounted for 32.2% of infections, bacteria for 29.8%, and atypical agents for 29.7%, with significant findings of *Staphylococcus aureus*, *Moraxella catarrhalis*, and *Mycoplasma pneumoniae*. Age-related analysis indicated a higher incidence of bacterial infections in infants and viral infections in preschool-aged children, with atypical pathogens being most prevalent in 3–5-year-olds. Gender-based analysis, adjusted for age, revealed similar overall pathogen presence; however, females were more susceptible to viral infections, while males were more prone to *Streptococcus pneumoniae*. Notably, there was an unusual increase in pathogen cases during spring, potentially influenced by behavioral changes and public health measures related to COVID-19. Co-infections were identified as a significant risk factor for the development of pneumonia.

**Conclusion:**

The study provides essential insights into the epidemiology of respiratory pathogens in pediatric populations, emphasizing the need for healthcare strategies tailored to age, gender, and seasonality. The findings highlight the impact of environmental and public health factors, including COVID-19 measures, on respiratory pathogen prevalence, underscoring the importance of targeted diagnostic and treatment protocols in pediatric respiratory infections.

## 1 Introduction

Respiratory tract infections (RTIs), including those caused by common pathogens such as the Respiratory Syncytial Virus (RSV), are prevalent worldwide and significantly contribute to illness and mortality in children under five years of age (1–3). For instance, in 2019, about 33.0 million episodes of RSV-associated acute lower respiratory infection (ALRI) were estimated globally in children aged 0–60 months, with an uncertainty range (UR) of 25.4–44.6 million. The in-hospital mortality from RSV-associated ALRI was estimated at 26,300 cases (UR: 15,100–49,100), and the total RSV-attributable deaths were approximately 101,400 (UR: 84,500–125,200) in this age group (2). RTIs are not only common reasons for seeking healthcare but also present significant challenges in clinical decision-making due to the complexity of their symptoms and management.

Building on the significant burden of RTIs in children, accurate pathogen detection is crucial for effective clinical management (4, 5). This precision in pathogen identification is key to avoiding unnecessary antimicrobial use, aligning treatments more closely with specific infections (6–8), thereby contributing to improved clinical outcomes in pediatric respiratory infections. In clinical diagnosis, RTIs present a significant challenge due to the nonspecific nature of their symptoms, which are often similar across various pathogens. This lack of specificity complicates the rapid and effective identification of the causative pathogen, potentially leading to delayed or inappropriate treatment. Such diagnostic challenges are particularly critical in children, where they can contribute to worsening health conditions (9). Moreover, the prevalence and significance of the pathogens can vary by geographical location, age groups, and study methodologies.

Regarding the research on epidemiological features of acute respiratory infections, the existing investigations primarily focus on specific regions in China like City of Zunyi (10), Chengdu (11), Xiamen (12), and Suzhou (13), or other countries’ specific regions such as Central Panama (14), and the findings may not be universally applicable. Only a few studies focus on the national level, such as China (15) and Senegal (16). Their investigations show that the main pathogens causing respiratory tract infections in children include bacteria, viruses, and atypical pathogens, with most infections in the respiratory population caused by viruses and atypical pathogens (16). Among them, the viruses with the highest detection rates are RSV, human rhinovirus (HRV), influenza A virus (IAV), influenza B virus (IBV), parainfluenza virus (PIVs), and adenovirus (ADV) (10, 11, 14). In Chinese national surveillance of patients with acute respiratory tract infections from 2009 to 2019 (15), the top three viral infections were influenza virus, RSV, and HRV; *Streptococcus pneumoniae* and *Klebsiella pneumoniae* were the top two pathogens; *Mycoplasma pneumoniae* (MP) was the main type of atypical pathogen infection.

In the context of the COVID-19 pandemic, there has been a noted increase in antibiotic prescribing without sufficient evidence of bacterial co-infections, contributing to the growing concern over antimicrobial resistance (AMR). This trend underscores the critical need for improved antimicrobial stewardship and targeted interventions to refine antibiotic prescribing practices for ARIs and upper respiratory tract infections (URTIs), mitigating the burgeoning threat of AMR. Studies such as those by Duffy et al. (17), which highlight that community antibacterial consumption comprises a significant portion of total antibacterial use, and by Godman B et al. (18), discussing strategies to reduce inappropriate antibiotic use, particularly in LMICs, emphasize the global scale of this challenge (17, 18). The excessive prescribing patterns observed during the pandemic, as reported by Mustafa ZU et al. (19) among neonates and children in Pakistan, further illustrate the urgent need for concerted efforts to address this issue (19).

Most studies have focused on the specific region is reasonable as the distribution and epidemiological characteristics of RTI pathogens could be moderately different due to the vast territory and diverse climate in different regions, as well as differences in sanitary conditions, the distribution of pathogen infections varies in different regions. Therefore, continued local epidemiological surveillance is necessary to obtain the latest information and to develop appropriate infection control and preventive intervention measures, particularly in western and southern region of China, which has not been fully explored on the epidemiological characteristics of acute respiratory pathogens in Children in recent years (11). In addition, epidemiology of respiratory tract infection could be changed by the outbreak of COVID-19 (20), highlighting the need for investigating the trends variation of pathogen detection among children. We also identified that there have been few reports that analyze atypical pathogens, viruses, and bacteria simultaneously; thus, a comprehensive and systematic investigation including a vast range of pathogens is needed. For example, in the study by Shi and Huang (13), while investigating the prevalence of respiratory pathogens and the risk of developing pneumonia under non-pharmaceutical interventions in Suzhou, China, the focus was predominantly on viral infections. This approach provides valuable insights into the viral landscape of respiratory infections in children; however, it falls short in addressing the coexistence and impact of bacterial pathogens.

This study conducted a comprehensive analysis of the spectrum and epidemiological characteristics of acute respiratory pathogens in children in southern Sichuan region, China from 2018 to 2021. Our choice of this specific study period is particularly pertinent due to the emergence of the COVID-19 pandemic at the beginning of 2020 in China. The global health crisis caused by the novel coronavirus has significantly altered the landscape of respiratory diseases, potentially impacting the prevalence and types of other RTIs. Our goal is to provide more accurate reference data and support for local clinical diagnosis, treatment, and prevention of respiratory diseases. Our region's distinct environmental, cultural, and healthcare attributes may influence the prevalence and types of RTIs, differing from global trends which predominantly focus on broader epidemiological data (21, 22). This regional specificity underscores the importance of our study in understanding and managing RTIs in this part of China, especially in the context of the evolving dynamics introduced by the COVID-19 pandemic.

The choice of Luzhou as the sample source region for this study is driven by two key reasons: Firstly, our hospital holds a representative position in the southern Sichuan area, with a high patient intake, ranking among the top in the region. Secondly, this study represents the first systematic and comprehensive analysis of pathogens in the Luzhou area, filling a research gap in this region. It uniquely combines indirect immunofluorescence and microbiological culture methods, simultaneously analyzing viruses, atypical pathogens, and bacteria. This approach aims to provide targeted insights into the local epidemiology of RTIs in the southern Sichuan region from 2018 to 2021, which could be crucial for developing region-specific health strategies and interventions.

The choice of Luzhou as the sample source region for this study is particularly relevant given the recent trends observed in the area. Notably, Luzhou has seen a significant incidence of *Mycoplasma pneumoniae*, a pathogen that often presents with subtle early symptoms like cough or sore throat, yet can lead to more severe respiratory conditions. This aspect of *Mycoplasma pneumoniae*, combined with its tendency for a prolonged disease course and potential for relapse, underscores the need for enhanced diagnostic and treatment approaches in the region. In the city of Luzhou, individuals with ARIs, including children, predominantly seek medical care directly from hospitals rather than community healthcare clinics or pharmacies. This preference reflects the public's trust in hospital-based care and the comprehensive services these institutions offer. Patients must pay for consultations at hospitals, which influences the decision to seek hospital care typically when symptoms of ARIs persist or worsen. Despite the costs, families prioritize accessing hospital-based services for more severe conditions, underscoring the value placed on obtaining accurate diagnoses and effective treatment. Outpatient individuals are typically required to cover the costs of pathogen testing out-of-pocket. These expenses, however, are relatively modest when evaluated against the income levels of the patient’s household. Conversely, hospitalized patients could receive a reimbursement for a specified portion of these costs through their medical insurance accounts. This affordability supports the implementation of targeted treatment strategies, reducing the reliance on empirical use of broad-spectrum antibiotics and contributing to more precise antimicrobial stewardship.

Our hospital, a leading healthcare provider in southern Sichuan, offers a unique vantage point for this study. It not only boasts a high patient intake but also provides a diverse patient profile, making it an ideal setting for a comprehensive analysis. By incorporating indirect immunofluorescence and microbiological culture methods to examine a spectrum of viruses, atypical pathogens, and bacteria, this study attempts to first provide detailed insights into the local epidemiology of RTIs from 2018 to 2021. The findings are expected to be pivotal in shaping region-specific health strategies, particularly in addressing the challenges posed by *Mycoplasma pneumoniae* and other significant respiratory pathogens in Luzhou.

## 2 Materials and methods

### 2.1 Participant selection

This retrospective study was conducted at the affiliated hospital of Southwest Medical University from July 2018 to January 2021 in city of Luzhou. We collected data on patients aged ≤14 years with respiratory tract infections (RTIs) through the Hospitalization Information System (HIS). RTI diagnoses were made based on the “Inpatient Severe Acute Respiratory Infection Case Surveillance Protocol (2011 Edition)” from the Chinese Center for Disease Control and Prevention. This protocol includes clinical criteria such as fever (body temperature ≥37.5°C), cough (dry or productive), sore throat, nasal congestion, runny nose, sinus pressure, respiratory distress (rapid breathing, hypoxia, shortness of breath, chest pain, discomfort, general breathlessness), and, where available, radiographic evidence of pneumonia (infiltrates, consolidation). These criteria were designed to accurately identify acute respiratory infections and ensure the inclusion of relevant cases in our study.

Exclusion criteria were applied to ensure the validity of our findings. We excluded patients readmitted within 30 days for the same RTI episode, cases with incomplete clinical data (missing demographic information, clinical history, symptomatology, illness duration, laboratory and radiographic findings, treatment details, and follow-up data), children with known immunodeficiencies or on immunosuppressive therapies, those with recent hospitalization or antibiotic use (within the past 3 months), and children with significant concurrent illnesses (e.g., congenital heart disease, renal failure). Additionally, children recently vaccinated against the pathogens of interest and patients enrolled in other clinical trials were excluded, along with cases where reliable medical history could not be obtained due to significant communication barriers, barring availability of translation or interpretation services.

The patients were categorized into four age groups: the infant group (0 to <1 year), the toddler group (1–3 years), the pre-school group (4–6 years), and the school-age group (7–14 years). Note the criteria for categorizing children into the distinct age groups in studies of respiratory infections are supported by various studies (10, 13, 14). IgM antibody tests for nine respiratory pathogens and MP antibody titer tests, bacterial cultures of sputum and/or throat swabs, and other specified etiological tests were conducted as part of the study. In accordance with China's climatic patterns, the four seasons were classified in this manner: Spring encompassed the months of March, April, and May; Summer included June, July, and August; Autumn was defined as the period from September to November; and Winter spanned December, January, and February of the subsequent year.

### 2.2 Instruments and reagents

In our study, the selection of instruments and reagents was guided by their proven effectiveness, reliability, and specificity in identifying respiratory pathogens. The culture media, staining solutions, and identification equipment were chosen for their high-quality properties, which are crucial for accurate bacterial growth, differentiation, and identification. The quality control strains were selected based on their well-established characteristics, ensuring the consistency of our culture processes. The diagnostic kit was chosen for its comprehensive coverage and high sensitivity in detecting respiratory infections, while the microscopy equipment was preferred for its superior optical and fluorescence capabilities, essential for detailed examination in immunofluorescence assays.

• Culture Media: Blood, MacConkey, and chocolate agar from Zhengzhou Antu Biological Engineering Co., Ltd.
• Staining Solutions: Gram staining solutions by Zhuhai Besso Biotechnology Co., Ltd.
• Identification Equipment: Microflex LT/SH microbial identification mass spectrometer and accessories from Bruker (Beijing) Technology Co., Ltd.
• Quality Control Strains: *Escherichia coli* ATCC25922, *Klebsiella pneumoniae* ATCC700603, *Staphylococcus aureus* ATCC25923, *Pseudomonas aeruginosa* ATCC27853, *Haemophilus influenzae* ATCC49247, *Streptococcus pneumoniae* ATCC29213, *Acinetobacter baumannii* ATCC196606 from Shanghai Baolu Biological Technology Co., Ltd.
• Diagnostic Kit: Respiratory nine-plex detection kit by Vircell, Spain (supplied by Zhengzhou Antu Biological Engineering Co., Ltd.).
• Microscopy: EUROStar III Plus fluorescence microscope from Germany

### 2.3 Data collection and analysis procedures

#### 2.3.1 Sample collection

Patient venous blood samples, approximately 2–3 ml each, were carefully drawn and deposited into standard separation tubes. Following collection, serum was promptly extracted and refrigerated at 4°C for preservation. All serum analyses were conducted within a 3-day window to ensure sample integrity.

Concurrently with blood sample collection, respiratory specimens were also gathered. Generally, most children over 3 years old in this study region can produce sputum through natural deep coughing. For those who cannot produce sputum or for younger children, electric sputum suction is used. Sputum, which is the primary type of specimen collected, is used for bacterial culture. This is supplemented by a small number of endotracheal tube (PICC) tip specimens. During the study period, there were very few alveolar lavage fluid and throat swab specimens, although the collection of alveolar lavage fluid specimens has increased in the past two years. Throat swab specimens are usually collected when the child's symptoms are mild or when sputum specimens are difficult to obtain. The sterile pharyngeal swab is placed in a sterile cup, and some may be dipped in a small amount of saline and sent for examination in a timely manner. Specimens were collected before the administration of antibiotics whenever possible to ensure accurate bacterial culture results. This approach was taken to prevent the suppression of bacterial growth, which could lead to false-negative results. All respiratory specimens, including sputum, throat swabs, and others, were processed within a strict 24 h timeframe to maximize the accuracy and reliability of the bacterial assessment.

#### 2.3.2 Antibody testing for respiratory pathogens

In this phase, the detection of IgM antibodies specific to nine respiratory pathogens in patient serum was carried out utilizing the indirect immunofluorescence technique. The targeted pathogens included *Legionella pneumophila* serogroup 1 (LP1), MP, *Q fever Coxiella* (COX), *Chlamydophila pneumoniae* (CPn), ADV, RSV, IAV, IBV, and (PIVs). The testing procedures were meticulously adhered to as per the manufacturer's guidelines for both the equipment and the test kits. For the assessment and interpretation of results, the EUROStar III Plus fluorescence microscope was employed, ensuring high precision and reliability in the antibody detection process.

It is important to note that the use of IgM antibody detection has inherent limitations. A positive antibody result does not necessarily indicate an acute infection but rather that the individual has been exposed to the pathogen at some point. This limitation is particularly relevant for pathogens that can cause asymptomatic or chronic infections. To address this, antibody testing was complemented with bacterial culture and identification techniques to provide a more comprehensive diagnostic approach. The combination of these methods aims to improve the accuracy of pathogen identification and support clinical decision-making. Despite its limitations, the comprehensive IgM detection included in this study covers a wide range of common pathogens, including viruses and bacterial agents like *Mycoplasma pneumoniae*, *Legionella pneumophila*, and *Rickettsia*, providing valuable information for understanding the patients’ exposure to various respiratory pathogens.

#### 2.3.3 Bacterial culture and identification

The process of bacterial isolation and culture was meticulously executed in strict adherence to established national clinical laboratory protocols. Following isolation, the pathogens were subjected to Gram staining, a critical step in the initial classification of the bacteria. Based on the outcomes of the Gram staining, appropriate identification cards were carefully selected to facilitate further analysis. The final step of bacterial identification was conducted with precision using the Microflex LT/SH automatic microbial mass spectrometer, a state-of-the-art instrument provided by Bruker. This sophisticated technology ensured an accurate and reliable identification of the bacterial species present in the samples.

To differentiate pathogenic bacteria from normal flora or colonizing organisms, particularly in throat swabs, several criteria were employed. The bacterial density was assessed, with higher densities being more indicative of pathogenic bacteria. Quantitative cultures were performed, and organisms present in higher concentrations were considered more likely to be pathogens. Culture results were interpreted in conjunction with clinical findings, such as signs and symptoms of infection, to determine the clinical relevance of the cultured organisms. Additionally, molecular techniques, such as PCR, were used to confirm the presence of specific pathogens, and serological tests were employed to detect antibodies against suspected pathogens, providing further evidence of infection. Specific criteria for identifying pathogens included the presence of typical respiratory pathogens, such as *Streptococcus pneumoniae*, *Haemophilus influenzae*, and *Staphylococcus aureus*, in significant quantities. Normal flora organisms were identified and documented but not considered pathogens unless present in high density and associated with clinical symptoms. These steps ensured a comprehensive approach to accurately distinguish between pathogenic bacteria and normal flora or colonizing organisms (23).

In the rare instances where both sputum and throat swab samples were collected from the same subject, bacterial cultures were performed on both types of specimens. If the culture results differed, priority was given to the sputum culture results.

### 2.4 Statistical analysis

The statistical analysis was a critical component of this retrospective study and was conducted using the SPSS software version 26.0. Our cohort comprised children aged ≤14 years presenting with respiratory tract infections (RTIs), stratified into four age-based cohorts: infant, toddler, pre-school, and school-age. Our analysis incorporated results from the IgM antibody test for nine respiratory pathogens, MP antibody titer tests, bacterial cultures from sputum and throat swabs, and a comprehensive etiological evaluation to determine the prevalence and distribution of respiratory pathogens across different age groups and seasons.

We utilized the *χ*^2^ test or Fisher's exact test to compare categorical data across the cohorts. For continuous data adhering to a normal distribution, the mean ± standard deviation (x ± s) was calculated, and comparative analysis between two groups was executed using the independent samples t-test. For multiple group comparisons, one-way ANOVA was the chosen method. Conversely, skewed data were presented as medians with interquartile ranges [M (P25, P75)], and non-parametric tests such as the Mann–Whitney *U* test and the Kruskal–Wallis H test were used for two-group and multiple group comparisons, respectively.

To control for age-related bias in gender comparisons of pathogen detection rates, Propensity Score Matching (PSM) was implemented. This approach allowed us to equalize age distributions between male and female participants and isolate gender as the independent variable of interest. Further, a binary logistic regression model was developed to explore risk factors for severe pneumonia in the pediatric subjects. This model utilized the forward selection method for the inclusion of variables, ensuring the robustness of the analysis.

All statistical tests were two-sided, with a *P*-value of <0.05 considered statistically significant. This threshold adheres to standard practices in medical research and allows for a rigorous determination of meaningful differences and trends in the data.

The study was conducted with the approval of the Ethics Committee of Southwest Medical University, referenced under the ethics approval number KY2022225.

## 3 Results

### 3.1 Demographic and pathogen distribution in pediatric respiratory infections

Our study encompassed a diverse group of 12,546 children suffering from RTIs. Within this cohort, 9,934 cases underwent IgM antibody tests for nine respiratory pathogens and MP antibody titer tests. Furthermore, 9,056 cases had bacterial cultures of sputum and/or throat swabs, and 6,444 cases completed all specified etiological tests. Among these 12,546 patients, males constituted 61% (7,654) while females accounted for 39% (4,892) with gender ratio of 1.56:1. The median age was 12 months, with an interquartile range from 4 to 36 months.

Pathogen detection was pivotal in our analysis (Table 1), with a detection rate of 52.8% (6,618/12,546). Through bacterial culture and identification conducted on 9,056 specimens, bacterial pathogens were identified in 29.7% of cases, with *Staphylococcus aureus* (7.3%) and *Moraxella catarrhalis* (6.1%) being the most prevalent. Viral infections were confirmed in 29.8% of 9,934 cases, with IBV and PIVs being the most detected, with 20.2% and 8.8% cases, respectively. Atypical pathogens, predominantly MP, were present in 32.2% of the cases examined. Co-infections, defined as the simultaneous presence of a combination of viruses, bacteria, or atypical pathogens in a single patient, were identified in 22.3% of the children. The most frequent combination found in co-infections involved atypical and viral pathogens.

**Table 1:** Pathogen profiles of children with RTI.

Category of pathogen	Identified pathogens	Number of cases (percentage)
Bacteria (2,692/9,056, 29.7%)	*Staphylococcus aureus*	663 (7.3%)
	*Moraxella catarrhalis*	550 (6.1%)
	*Haemophilus influenzae*	473 (5.2%)
	*Streptococcus pneumoniae*	283 (3.1%)
	*Escherichia coli*	246 (2.7%)
	*Klebsiella pneumoniae*	222 (2.5%)
Virus (2,960/9,934, 29.8%)	ADV	197 (2.0%)
	RSV	517 (5.2%)
	IAV	191 (1.9%)
	IBV	2,002 (20.2%)
	PIVs	872 (8.8%)
Atypical pathogens (3,197/9,934, 32.2%)	LP-1	81 (0.8%)
	MP	3,122 (31.4%)
	COX	29 (0.3%)
	CPn	43 (0.4%)
Co-infection (1,434/6,444, 22.3%)	Atypical Pathogen + Virus	710 (11.0%)
	Atypical Pathogen + Bacteria	224 (3.5%)
	Virus + Bacteria	314 (4.9%)
	Bacteria + Virus + Atypical Pathogen	186 (2.7%)

### 3.2 Age-related variations in pathogen detection among children

**Figure 1:**
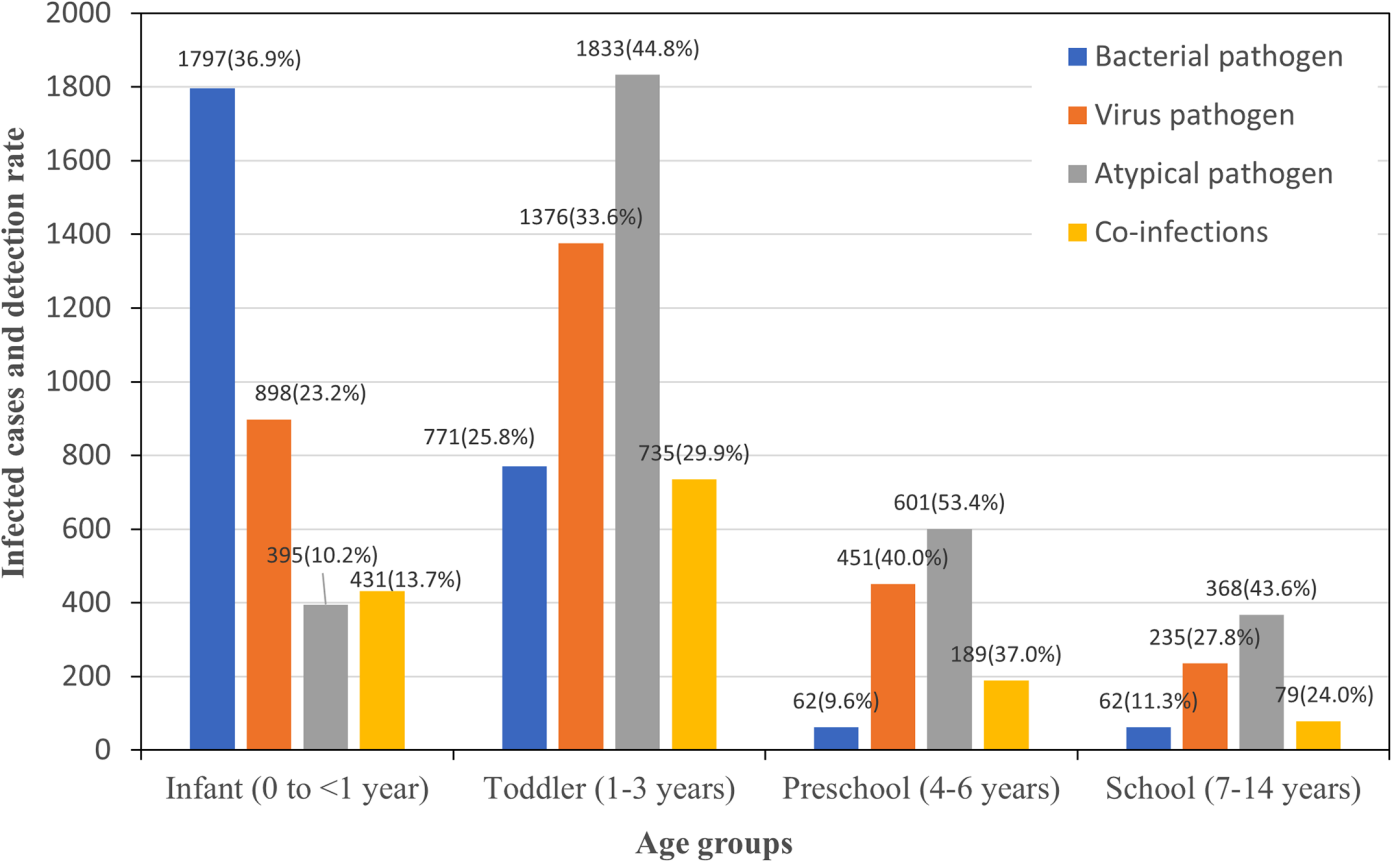
Pathogen detection among ARTs children at different age groups.

The comprehensive data presented in Figure 1 and Table 2 underscore statistically significant differences in the detection of main pathogens across pediatric age groups. Each *χ*^2^ or *P* value marked with an asterisk indicates that these differences are statistically significant, with *P* < 0.05.

**Table 2:** Comparison of pathogen detection across different age groups.

Category	Infant group (*n* = 5,585)	Toddler group (*n* = 4,631)	Preschool group (*n* = 1,267)	School age group (*n* = 1,063)	*χ*^2^ or *P*
Serum antibody test (cases)	3,871	4,091	1,127	845	–
Bacterial culture and identification (cases)	4,868	2,991	647	550	–
Bacterial pathogen (detection rate)					
*Staphylococcus aureus*	538 (11.05%)	95 (3.18%)	13 (2.01%)	17 (3.09%)	2,167.0*
*Moraxella catarrhalis*	250 (5.14%)	285 (9.52%)	11 (1.70%)	4 (0.73%)	119.4*
*Haemophilus influenzae*	272 (5.59%)	190 (6.35%)	5 (0.78%)	6 (1.09%)	53.9*
*Streptococcus pneumoniae*	149 (3.06%)	120 (4.01%)	13 (2.01%)	1 (0.18%)	26.2*
*Escherichia coli*	209 (4.29%)	32 (1.07%)	3 (0.46%)	2 (0.36%)	100.4*
Virus pathogen (detection rate)					
RSV	385 (9.95%)	110 (2.69%)	15 (1.33%)	7 (0.83%)	295.9*
ADV	50 (1.29%)	100 (2.44%)	37 (3.28%)	10 (1.18%)	26.6*
IBV	397 (10.26%)	1,030 (25.18%)	366 (32.48%)	210 (24.85%)	417.6*
PIVs	240 (6.20%)	424 (10.36%)	138 (12.24%)	70 (8.28%)	62.2*
Atypical pathogen (detection rate)					
LP-1	7 (0.18%)	50 (1.22%)	11 (0.99%)	13 (1.54%)	33.5*
MP	382 (9.87%)	1,798 (43.95%)	593 (53.33%)	349 (41.30%)	1,405.6*
All Pathogen Testing (cases)		3,147	2,457	511	329

*Statistically significant difference (*P* < 0.05).

For bacterial pathogens, infants showed the highest susceptibility, with a total detection rate of 36.91%, including notably high incidences of *Staphylococcus aureus* at 11.05%. As age increased, there was a marked decrease in the overall bacterial detection rate, with the lowest observed in the preschool group at 9.58%. Viral infections presented a different trend, with the lowest detection rate in infants at 23.20% and a peak in the preschool group at 40.00%. Notably, RSV was most commonly detected in infants at 9.95%, with its incidence diminishing with age, while IBV's rate generally increased in younger age groups before school age. In addition, IBV had a significant presence across all age groups, particularly high in the preschool group at 32.48%. The detection rate for atypical pathogens was highest in the preschool group at 53.43%, with MP as the predominant pathogen, indicating a peak in these infections among children aged 3–5 years. Co-infections were identified in 22.3% of the cases where all pathogen tests were conducted, with the highest rate found in the preschool group at 36.99%, followed by the toddler group at 29.91%. This suggests that the likelihood of simultaneous infections from different pathogen categories increases with age up to the preschool years, after which it slightly declines.

The *χ*^2^ or *P* values, particularly those with an asterisk, underscore the statistical significance of the variations across age groups, confirming that the distribution and prevalence of pathogens are age-dependent. These findings highlight the need for age-specific diagnostic and treatment approaches in managing pediatric infections.

### 3.3 Gender-related variations in pathogen detection among children

To ensure a rigorous and unbiased comparison of pathogen prevalence across genders, it was imperative to adjust for age bias. Age can be a confounding factor due to differential exposure and immune system maturity in children of different ages. Consequently, we employed age-bias matching by Propensity Score Matching to align the demographic distribution, thus isolating gender as the variable of interest. After this adjustment, the study involved an equal number of male and female participants (*n* = 4,892 for each gender), allowing for an equitable evaluation of pathogen detection rates (Table 3). The overall pathogen detection rates after age-bias matching were 56.5% for males and 55.5% for females, showing no significant difference (*χ*^2^ = 0.956, *P* > 0.05), indicating that gender does not play a significant role in the presence of pathogens when age distribution is accounted for.

**Table 3:** Detection of pathogens in children with RTI across different genders after age-bias matching.

Category	Male	Female	*χ* ^2^
Number of cases	4,892	4,892	–
Total pathogen detection rate	2,764 (56.5%)	2,716 (55.5%)	0.956
Serum antibody test (cases)	3,885	3,885	–
Virus pathogen (detection rate)			
ADV	72 (1.9%)	72 (1.9%)	0.000
RSV	290 (7.5%)	189 (4.9%)	22.696*
IBV	699 (18.0%)	945 (24.3%)	46.610*
PIVs	149 (3.8%)	435 (11.2%)	151.444*
Atypical pathogen (detection rate)			
LP-1	20 (0.6%)	37 (1.0%)	5.108*
MP	1,107 (28.5%)	1,509 (38.8%)	93.130*
Bacterial culture and identification (cases)	3,454	3,454	–
Bacterial pathogen (detection rate)			
*Staphylococcus aureus*	252 (7.3%)	235 (6.8%)	0.638
*Moraxella catarrhalis*	181 (5.2%)	219 (6.3%)	3.832
*Haemophilus influenzae*	193 (5.6%)	175 (5.1%)	0.930
*Streptococcus pneumoniae*	154 (4.5%)	96 (2.8%)	13.961*
*Escherichia coli*	94 (2.7%)	95 (2.8%)	0.005
*Klebsiella pneumoniae*	91 (2.6%)	80 (2.3%)	0.726
All pathogen testing (cases)	2,448	2,448	–

*Statistically significant difference (*P* < 0.05).

**Figure 2:**
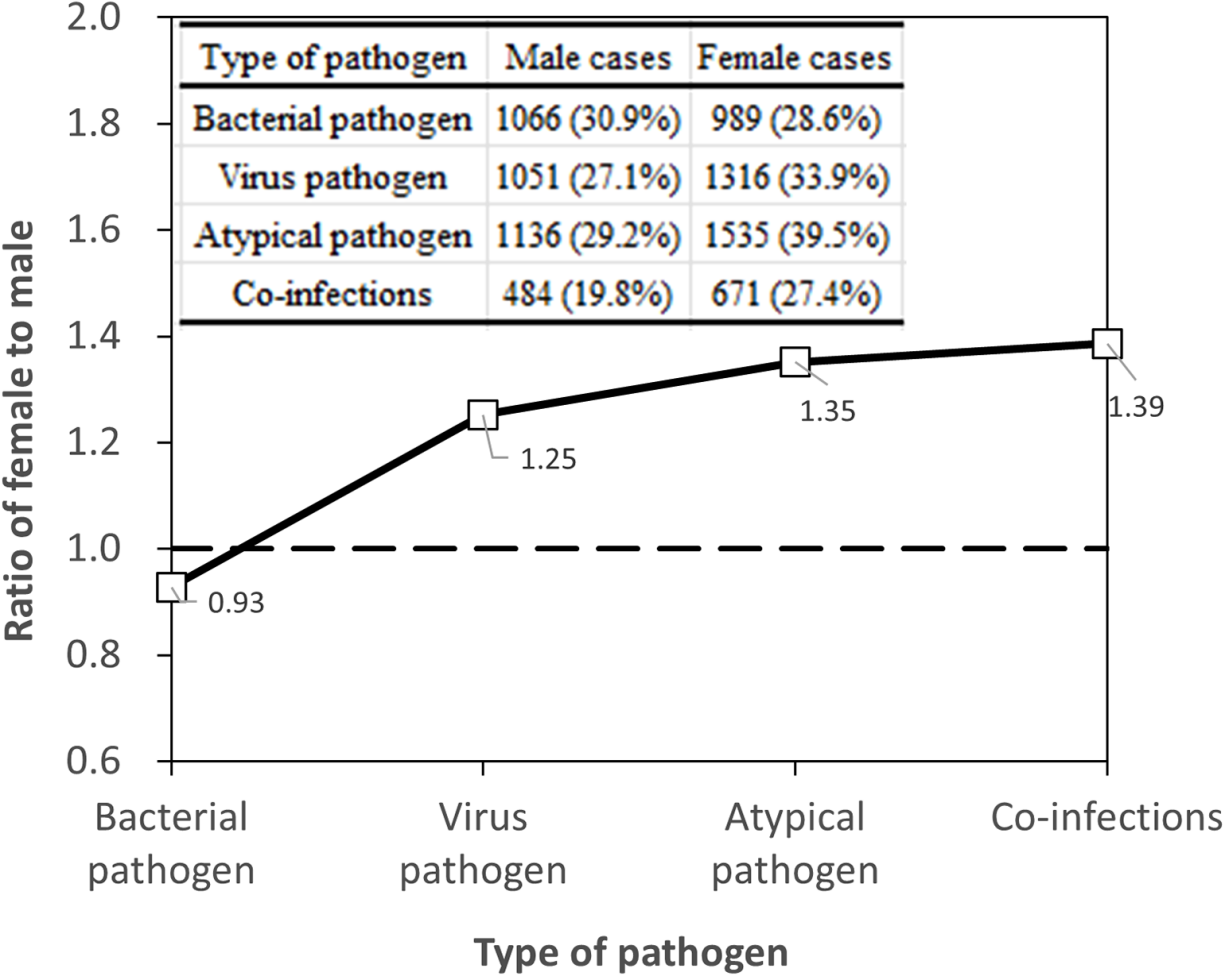
Gender-specific pathogen prevalence: female-to-male case ratio across pathogen types.

Serum antibody testing (*n* = 3,885 for each gender) yielded a total virus detection rate of 27.1% for males and 33.9% for females. The difference remained statistically significant (*χ*^2^ = 42.666*, *P* < 0.05), with females showing a higher prevalence of viral infections, as shown in Figure 2. Specifically, the detection rates for RSV decreased from 7.5% in males to 4.9% in females (*χ*^2^ = 22.696*), and for IBV from 18.0% in males to 24.3% in females (*χ*^2^ = 46.610*), post age-bias matching. Atypical pathogen detection rates were 29.2% for males and 39.5% for females, with a significant difference (*χ*^2^ = 90.826*, *P* < 0.05). MP remained the most prevalent atypical pathogen, with a higher incidence in females (38.8%) compared to males (28.5%). For bacterial pathogens, the overall detection rates were 30.9% for males and 28.6% for females after matching, with a slight but significant difference (*χ*^2^ = 4.107*, *P* < 0.05). *Streptococcus pneumoniae* detection was notably higher in males (4.5%) compared to females (2.8%) post matching, signifying statistical significance (*χ*^2^ = 13.961*, *P* < 0.05). The co-infection detection rates post age-bias matching were 19.8% for males and 27.4% for females at female-to-male case ratio of 1.39 (Figure 2), confirming a higher incidence of co-infections in females (*χ*^2^ = 39.624*, *P* < 0.05).

These post-matching statistics underscore the importance of age as a confounding factor in gender comparisons and highlight the need for age-adjusted analysis in epidemiological studies of pediatric respiratory tract infections.

### 3.4 Seasonal variation in pathogen prevalence among pediatric RTI cases

**Figure 3:**
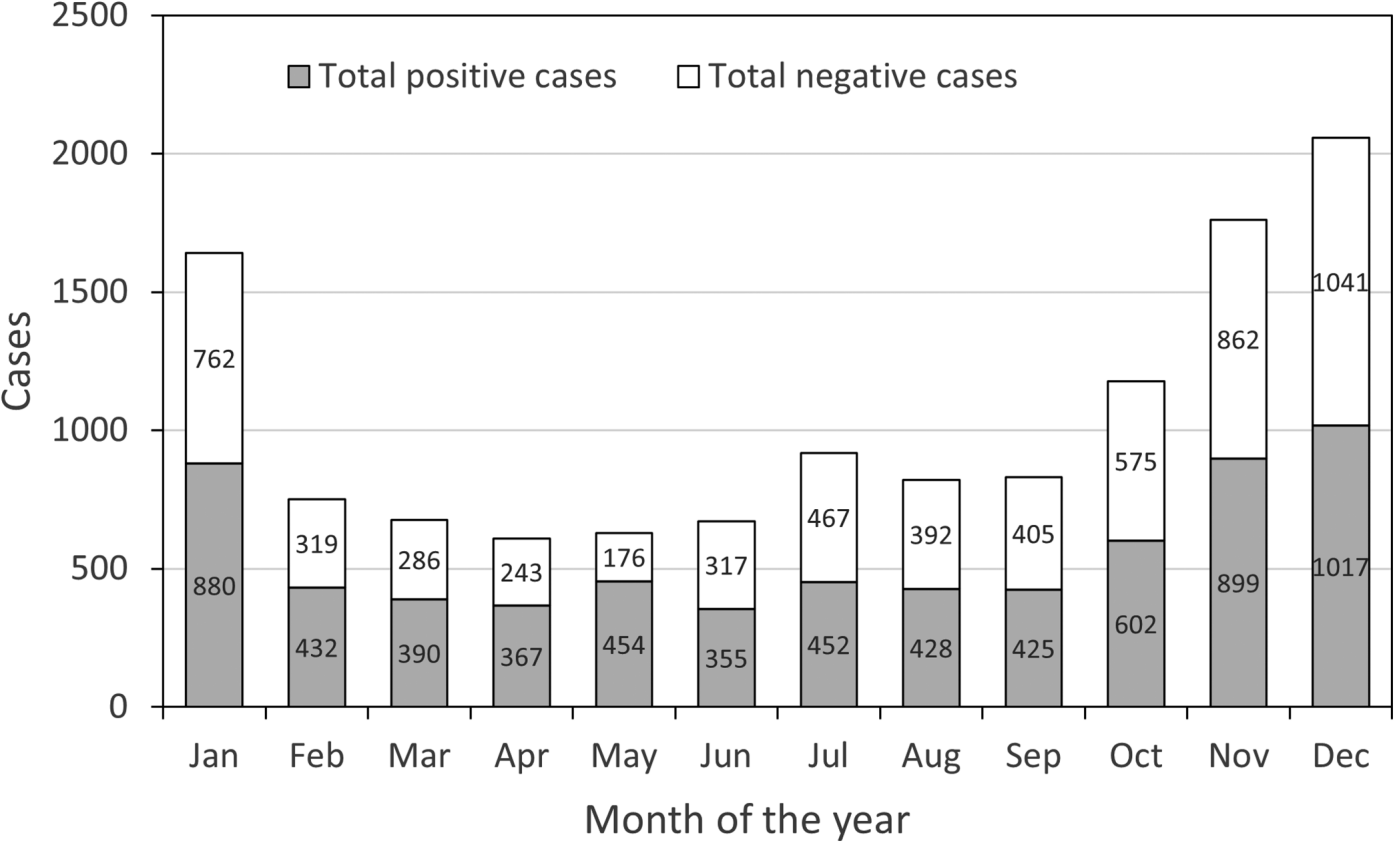
Number of total positive and negative cases of ARTs children in different months from January to December.

The monthly data from the study reveal a discernible pattern in the occurrence of RTIs in children, differentiated by the number of positive and negative cases, as shown in Figure 3. Notably, there is a clear increase in the number of positive cases towards the latter part of the year, with December marking the highest number at 1,017 cases, suggesting a seasonal peak during the colder months. This could be attributed to factors such as increased indoor congregation, which facilitates the transmission of respiratory pathogens. On the other hand, the number of negative cases remains relatively consistent throughout the year, with a slight increase also observed in December, indicating that while the infection rate increases, the overall testing rate might also rise during this month.

Delving into the specifics provided by Table 4, the seasonal analysis of pathogen detection offers a detailed understanding of how different pathogens exhibit varying prevalence throughout the year. In the spring, there is a high rate of viral detection at 37.8%, with IBV and atypical pathogens like MP being particularly prominent. The prevalence of IBV continues into the summer, maintaining a detection rate of 28.8%, alongside an increased detection of PIVs. Autumn presents a reduced viral detection rate overall, yet it stands out as the season with the highest detection rate for PIVs at 12.1%. As winter arrives, there is a significant dip in the overall viral detection rate to 23.8%; however, RSV detection increases to 5.5%, which aligns with the typical seasonal incidence of RSV. The bacterial pathogen detection rate also shows seasonal variation, with winter having the highest rate at 34.9%. This season sees a substantial rise in *Haemophilus influenzae* detections, suggesting an increased vulnerability to bacterial respiratory infections during colder weather. Co-infection rates are highest in spring at 30.0% and gradually decrease to the lowest point in winter at 18.8%. This trend indicates that while individual pathogen prevalence may vary, the overall risk of co-infection is greater during seasons with higher pathogen circulation.

**Table 4:** Comparison of pathogen detection rate in different seasons.

Category	Spring (*n* = 1,916)	Summer (*n* = 2,411)	Autumn (*n* = 3,768)	Winter (*n* = 4,951)	*χ* ^2^
Serum antibody test (cases)	1,477	1,842	3,009	3,606	–
Total virus (detection rate)	559 (37.8%)	651 (35.3%)	890 (29.6%)	859 (23.8%)	134.480*
RSV	108 (7.3%)	94 (5.1%)	116 (3.9%)	199 (5.5%)	25.164*
ADV	32 (2.2%)	42 (2.3%)	51 (1.7%)	72 (2.0%)	2.381
IBV	426 (28.8%)	530 (28.8%)	591 (19.6%)	455 (12.6%)	282.086*
PIVs	80 (5.4%)	150 (8.1%)	364 (12.1%)	278 (7.7%)	68.309*
Atypical pathogen (detection rate)	599 (40.6%)	698 (37.9%)	955 (31.7%)	944 (26.1%)	134.819*
LP-1	9 (0.6%)	11 (0.6%)	27 (0.9%)	34 (0.9%)	2.834
MP	591 (40.0%)	683 (37.1%)	932 (31.0%)	915 (25.4%)	139.448*
Bacterial culture and identification (cases)	1,426	1,463	2,652	3,515	–
Total bacterial (detection rate)	408 (28.6%)	337 (23.0%)	722 (27.2%)	1,225 (34.9%)	84.335*
*Staphylococcus aureus*	103 (7.2%)	81 (5.5%)	180 (6.8%)	299 (8.5%)	15.279*
*Moraxella catarrhalis*	59 (4.1%)	57 (3.9%)	195 (7.4%)	239 (6.8%)	32.387*
Haemophilus influenzae	77 (5.4%)	26 (1.8%)	77 (2.9%)	293 (8.3%)	135.879*
*Streptococcus pneumoniae*	43 (3.0%)	22 (1.5%)	80 (3.0%)	138 (3.9%)	20.312*
*Escherichia coli*	40 (2.8%)	36 (2.5%)	67 (2.5%)	103 (2.9%)	1.375
*Klebsiella pneumoniae*	36 (2.5%)	54 (3.7%)	50 (1.9%)	82 (2.3%)	13.193*
All pathogen testing (cases)	983	896	1,854	2,711	–
Co-infection (detection rate)	292 (29.7%)	236 (26.3%)	393 (21.2%)	510 (18.8%)	62.581*
Category	Spring (*n* = 1,916)	Summer (*n* = 2,411)	Autumn (*n* = 3,768)	Winter (*n* = 4,951)	*χ* ^2^

*Statistically significant difference (*P* < 0.05).

In summary, the monthly and seasonal data combined underscore the need for heightened vigilance and preparedness for RTIs in children, particularly during the winter months. These patterns should guide healthcare planning and preventative measures to mitigate the impact of seasonal infections.

### 3.5 Correlation between pathogen presence and severity of pediatric respiratory infections

The severity of the RTIs was categorized based on clinical assessment. Mild cases, including bronchitis and other upper respiratory tract infections, constituted 29.5% (3,706 children) of the cohort. The majority of cases were deemed moderate, typically presenting as pneumonia, and accounted for 60.1% (7,538 children) of the cohort. Severe pneumonia, requiring more intensive medical interventions, comprised 7.0% (882 children).

Analysis of pathogen detection rates among children with RTIs of varying severity revealed significant disparities. With the increase in disease severity, there was a noted increase in the detection rates of bacteria, atypical pathogens, and overall pathogens. The statistical significance of the differences in virus and atypical pathogen detection rates highlights MP and IBV as the primary pathogens associated with the onset and progression of RTIs to pneumonia in children in the southern Sichuan area. The median age of children tended to decrease with the increasing severity of the disease, implying that younger children may be more vulnerable to severe RTIs. Gender distribution across the groups showed more males than females; however, the difference was not statistically significant (*χ*^2^ = 5.240, *P* > 0.05).

The overall detection rate of pathogens correlated with disease severity, being 44.6% in the mild group, 55.9% in the moderate group, and peaking at 56.3% in the severe group (*χ*^2^ = 132.090*). This correlation was consistent with the virus detection rate, where the mild group exhibited a 24.6% rate, the moderate group 31.1%, and the severe group 43.8% (*χ*^2^ = 92.137*).

RSV and ADV showed a progressive increase in detection rates correlating with disease severity. However, IBV had a notably high rate in the severe group at 32.3% (*χ*^2^ = 56.240*), indicating its significant role in exacerbating RTIs. PIVs were more commonly detected as the severity of the disease increased, peaking at 11.8% in the severe group. Atypical pathogens, with MP leading, marked a substantial rise from 29.3% in the mild group to 44.0% in the severe group, accentuating their stronger prevalence in more severe cases (*χ*^2^ = 44.055*).

Upon assessing bacterial pathogens, an increase in detection rates was noted alongside the increase in RTI severity, reaching 38.0% in the severe group (*χ*^2^ = 98.143*). Notably, *Staphylococcus aureus* and *Klebsiella pneumoniae* saw increases, with the latter being significantly present in the severe group at 4.9% (*χ*^2^ = 32.961*). Co-infections were indicative of disease severity, with detection rates of 14.7% in the mild group, 23.6% in the moderate group, and the highest in the severe group at 32.9% (*χ*^2^ = 68.530*).

The observed trend indicates that co-infections may act as a risk factor for the development of pneumonia from acute upper respiratory tract infections. The heightened co-infection detection rate in the moderate group, significantly more than in the mild group, underscores the potential for acute upper respiratory infections to progress to pneumonia when multiple pathogens are involved. These findings underscore the necessity for thorough pathogen screening in the diagnosis and management of pediatric RTIs, with particular attention to the possibility of co-infections contributing to disease severity.

To assess whether co-infections act as an independent risk factor for the development of pneumonia from acute upper respiratory tract infections, a binary logistic regression model was established. The severity of pneumonia (respiratory tract infection = 0, pneumonia = 1) was used as the dependent variable, while gender (male = 0, female = 1), age, and co-infection (no = 0, yes = 1) were included as independent variables. The model included 3,726 children with positive pathogen detection, among which 839 had upper respiratory tract infections and 2,887 had pneumonia; 2,240 were male and 1,486 were female; 1,293 had co-infections, and 2,433 had no co-infections.

The logistic regression modeling results, as shown in Table 5, further substantiates the findings from Table 6, where the severity of respiratory tract infections correlated with an increase in pathogen detection rates. The regression analysis, which included 3,726 children with positive pathogen detection, revealed that co-infections pose a significant independent risk factor for the development of pneumonia. The multivariate analysis, accounting for age and gender, confirmed that co-infections nearly double the odds of pneumonia in affected children (OR = 1.940, *P* < 0.001). These results imply that in the clinical management of pediatric respiratory infections, identifying and addressing co-infections is crucial for preventing the progression to more severe diseases such as pneumonia.

**Table 5:** Multivariable logistic regression analysis of risk factors for pneumonia development from acute upper respiratory tract infections.

Variable	Coefficient (β)	Standard error (SE)	Wald *χ*^2^	Odds ratio (OR) [95% CI]	*P*-value
Gender (Male = 0, Female = 1)	0.034	0.082	0.174	1.035 [0.881–1.215]	0.677
Age (months)	−0.010	0.001	51.789	0.990 [0.987–0.993]	<0.001
Co-infection (No = 0, Yes = 1)	0.663	0.062	53.538	1.940 [1.624–2.316]	<0.001

**Table 6:** Detection of pathogens with different degrees of disease severity.

Category	Mild group (*n* = 3,706)	Moderate group (*n* = 7,538)	Severe group (*n* = 882)	*χ*^2^ or Hc
Age (months)	24 (11,48)	11 (3,24)	7 (1,24)	975.724*
Gender (Male/Female)	2,272/1,434	4,640/2,898	508/374	5.240
Total pathogen (detection rate)	1,653 (44.6%)	4,211 (55.9%)	497 (56.3%)	132.090*
Serum antibody testing (cases)	3,153	5,980	493	
Total virus (detection rate)	775 (24.6%)	1,858 (31.1%)	216 (43.8%)	92.137*
RSV	82 (2.6%)	383 (6.4%)	26 (5.3%)	61.747*
ADV	50 (1.6%)	116 (1.9%)	25 (5.1%)	26.787*
IBV	560 (17.8%)	1,209 (20.2%)	159 (32.3%)	56.240*
PIVs	253 (8.0%)	516 (8.6%)	58 (11.8%)	7.624*
Atypical pathogen (detection rate)	923 (29.3%)	1,941 (32.5%)	217 (44.0%)	44.055*
LP-1	25 (0.8%)	48 (0.8%)	4 (0.8%)	0.003
MP	904 (28.7%)	1,892 (31.6%)	213 (43.2%)	42.969*
Bacterial culture and identification (cases)	1,991	6,130	635	
Total bacterial (detection rate)	424 (21.3%)	1,934 (31.5%)	241 (38.0%)	98.143*
*Staphylococcus aureus*	99 (5.0%)	469 (7.7%)	60 (9.4%)	21.525*
*Moraxella catarrhalis*	133 (6.7%)	380 (6.2%)	22 (3.5%)	8.959*
*Haemophilus influenzae*	63 (3.2%)	377 (6.2%)	27 (4.3%)	28.123*
*Streptococcus pneumoniae*	34 (1.7%)	225 (3.7%)	11 (1.7%)	23.557*
*Escherichia coli*	39 (2.0%)	185 (3.0%)	19 (3.0%)	6.367*
*Klebsiella pneumoniae*	21 (1.1%)	162 (2.6%)	31 (4.9%)	32.961*
All pathogen testing (cases)	1,438	4,572	246	
Co-infection (detection rate)	212 (14.7%)	1,081 (23.6%)	81 (32.9%)	68.530*

*Statistically significant difference (*P* < 0.05).

## 4 Discussion

In this study, we executed a thorough retrospective analysis of pathogen prevalence among children with respiratory tract infections in the southern Sichuan region of China. Encompassing a wide array of pathogens, including viruses, bacteria, and atypical agents, our objective was to provide valuable epidemiological data to aid in the effective prevention and management of respiratory infections among local pediatric populations. Furthermore, our investigation sought to contribute to the optimization of medical resource utilization and the development of more targeted treatment modalities based on etiological findings. Our results revealed an overall pathogen detection rate of 52.8%, a figure that contrasts with the higher detection rates observed in studies involving hospital-admitted children with more severe RTIs. For instance, detection rates reported were 58.7% in 10,123 specimens from hospitalized patients at the Children's Hospital of Fudan University, Shanghai, China (24), and 72.7% at the Children's Hospital of Soochow University, Suzhou, China (13). As expected, these disparities highlight the variability of pathogen prevalence in relation to disease severity and geographical location. Regarding the detection rates of main pathogens, our results found the prevalence of viruses, bacteria, and atypical agents were detected in 32.2% (3,197/9,934), 29.8% (2,960/9,934), and 29.7% (2,692/9,056), respectively. These figures are of high variability compared to those from other studies (10, 11, 25) considering factors such as geographical location, meteorological factor, age group, sample inclusion criteria, the study period, and diagnostic methods. These various factors would cause a challenging data comparison with different studies.

The results of our study, after implementing age-bias matching through Propensity Score Matching, reveal intriguing insights into gender-related variations in pathogen detection among children. Notably, the overall pathogen detection rates showed no significant difference between males (56.5%) and females (55.5%), suggesting that gender does not significantly influence pathogen presence when age is considered. This is consistent with the investigation of the prevalence of respiratory pathogens in existing studies (12, 13, 26). However, gender disparities emerged in specific pathogen types. Females exhibited a higher prevalence of viral infections, particularly in IBV and PIVs, as well as a notably higher rate of atypical pathogens like MP. Conversely, males showed a higher detection rate for *Streptococcus pneumoniae*. These variations may be attributed to differences in immune system responses, hormonal influences, or even behavioral factors specific to each gender. Biological sex differences, including distinctions in sex chromosomes, reproductive tissues, and sex steroid concentrations, significantly impact immune cell function and immune responses. Females generally exhibit stronger type I and II interferon signaling and more robust humoral responses than males, which may explain their higher prevalence of certain infections (27). These sex differences in both innate and adaptive immunity contribute to the observed variations in pathogen prevalence and disease outcomes. The higher incidence of co-infections in females, as indicated by our findings, further underscores the complexity of gender differences in pediatric respiratory infections. These results highlight the critical role of age-adjusted analysis in understanding the epidemiology of respiratory tract infections in children and point to the need for further research into the underlying biological mechanisms driving these gender-specific patterns.

Our research identified RSV and IBV as primary respiratory viruses, which is in line with global patterns observed in studies such as those conducted in Suriname (28). However, contrasting patterns are evident in research from Suzhou, China (13), and Gabon (29), where HRV and MP, and AdV and PIV are more prevalent, respectively. This highlights the varied epidemiological profiles of respiratory viruses across different global regions and populations. Consequently, continuous surveillance is essential to grasp the changing dynamics of respiratory virus prevalence worldwide. In addition, it is important to consider the phenomenon of viral interference, particularly between epidemics of influenza and RSV as observed in previous studies (30, 31). This interference may also extend to other respiratory viruses like rhinovirus, coronavirus, and adenovirus. While our data could not confirm this due to limited numbers, the concept of viral interference—where the presence of one virus inhibits the activity or replication of another—is crucial in understanding the complex dynamics of respiratory virus prevalence. Factors like the production of interferon and cytokines by infected cells play a role in this interference (32).

The observed contrasting age-related trends in RSV and IBV prevalence in our study reflect age-specific susceptibility and immune responses to these viruses. The higher prevalence of RSV in infants can be attributed to their immature immune systems, which are less equipped to handle viral infections effectively (33). This vulnerability is compounded by the close physical contact infants typically have with caregivers, increasing their exposure to RSV (34). In contrast, the rise in IBV prevalence among older, preschool-age children may be due to increased social interactions, such as attending school, which heightens their exposure to the virus. Additionally, the maturation of the immune system in this age group might alter their response to IBV (35). These findings underscore the necessity of age-specific approaches in understanding and managing pediatric respiratory infections, considering the different immune competencies at various ages.

The high risk for children under 5 years and the notable increase in influenza virus infections in school-aged children (36) align with the observed age-related patterns in our study. These patterns may be influenced by several factors, including the development of immunity, social behaviors, and environmental exposures. For instance, the peak in viral detections, especially for atypical pathogens during the preschool years, might be linked to increased social interactions and attendance in communal settings like schools (37, 38). These environments can heighten exposure to various pathogens, contributing to the observed trends. This is particularly evident in the case of influenza virus infections, which are more prevalent among school-aged children, potentially due to more extensive social contacts and less mature immune systems compared to adults. Concurrently, the lower rates of viral infections in infants could be attributed to their limited external contacts and reduced opportunities for cross-infection, coupled with their developing immune systems, characterized by lower immunoglobulin levels. Interestingly, our findings also indicate a distinct pattern for bacterial infections, with infants being more susceptible, especially to infections caused by *Staphylococcus aureus*. These insights into age-specific trends in respiratory infections emphasize the need for targeted approaches in managing these infections in children of different age groups.

Influenza viruses, human coronaviruses, and respiratory syncytial viruses are often referred to as “winter viruses” due to their peak prevalence in the colder months (39). Contrary to typical epidemiological patterns that show influenza virus prevalence in winter and spring (13, 40), our study indicates a more pronounced prevalence in spring and summer for IBV and MP, as depicted in Table 4. This unexpected predominance in spring may be influenced by the post-winter resurgence of social activity and movements, potentially reducing the effectiveness of herd immunity gained during winter. The spring peak, as suggested by our findings, could also coincide with children spending more time indoors due to transitional weather, thereby facilitating viral transmission. Additionally, the lower infection rate observed in winter could be a result of effective public health measures, particularly the strict COVID-19 pandemic measures. Indeed, when comparing pre-pandemic data in our study, there was a noticeable decrease in both viral infection detection rates, by 2.9%, and co-infection detection rates, by 4.4%, in 2020 following the onset of the COVID-19 epidemic, consistent with existing studies (41). Therefore, the seasonal shifts in pathogen prevalence highlight the intricate dynamics of host-virus interactions and the impact of behavioral and environmental factors on disease transmission. The decrease in infection rates during the COVID-19 pandemic highlights the effectiveness of measures like enhanced hygiene and social distancing. Recognizing these seasonal dynamics can guide the timing of interventions and surveillance efforts, aiding in controlling respiratory pathogen spread and preparing for future pandemics.

MP is now recognized as the predominant atypical pathogen in pediatric RTIs, with a significant detection rate of 31.4%, hugely higher than other atypical pathogens (LP-1, COX and CPn). Our analysis reveals a marked prevalence of MP among preschool-aged and toddler demographics, with a higher incidence in females. Despite COVID-19 protective measures, the persistence of MP infections, particularly in southern Sichuan during the summer, highlights the need for vigilant monitoring of MP and SARS-CoV-2 co-infections to mitigate the risk of severe clinical outcomes (42, 43).

Our study's co-infection rate of 22.3%, predominantly involving atypical pathogens and viruses, underlines their critical role in progressing acute respiratory infections to pneumonia. This phenomenon is evident in the substantial prevalence of moderate cases like bronchopneumonia and pneumonia, with the moderate group showing a remarkable 4,572 cases. Logistic regression confirms that co-infections are indeed a risk factor, doubling pneumonia risk among children. It is essential for clinical management to prioritize the treatment of MP and co-infections to prevent deterioration. Future research could benefit from exploring vaccination strategies to mitigate co-infections’ impact on disease severity.

Despite the comprehensive nature of our retrospective analysis, several limitations warrant attention. Our study’s insights are primarily confined to the southern Sichuan region, limiting their broader applicability. The retrospective design precludes causality determination, and the overlap with the COVID-19 pandemic era may have influenced respiratory pathogen patterns atypically. Additionally, despite measures such as Propensity Score Matching to mitigate age-related biases, unaccounted variables could still influence outcomes. Notably, gender-based disparities in pathogen prevalence observed in our study call for further exploration into contributory biological and social factors. The use of diagnostic techniques less sensitive than PCR may have led to an underestimation of pathogen detection rates. Furthermore, the absence of long-term follow-up limits our understanding of the enduring effects of these infections. The study's omission of vaccination status is another factor that could skew the interpretation of pathogen prevalence and impact.

Additionally, although the use of IgM antibody detection has inherent limitations, as a positive antibody result does not necessarily indicate an acute infection but rather that the individual has been exposed to the pathogen at some point, we complemented this approach with bacterial culture and identification techniques to provide a more comprehensive diagnostic evaluation.

To address these limitations and enhance our understanding of pediatric respiratory infections, future research should focus on several key areas. Advanced diagnostic techniques, such as PCR, should be explored to improve the accuracy of pathogen detection. Longitudinal studies would provide valuable insights into the long-term outcomes of these infections. Investigating the impact of vaccinations on pathogen prevalence and the course of respiratory infections would also be beneficial. Additionally, a more in-depth examination of gender-based disparities, including the roles of biological and social factors, is crucial. Such research endeavors will not only address the current study's limitations but also significantly contribute to the effective management and understanding of respiratory infections in pediatric populations.

Our research into the clinical management of respiratory tract infections within the pediatric population of the southern Sichuan region not only enriches the diagnostic toolkit of healthcare professionals but also lays the groundwork for impactful public health strategies. Through our detailed profiling of prevalent respiratory pathogens and their epidemiological trends, we've established a data-driven foundation that enables the swift and precise identification of these pathogens, facilitating the implementation of customized treatment plans. More significantly, our findings carry profound implications for the realm of public health, particularly through the potential development of targeted immunization schedules and the strategic deployment of infection prevention initiatives. The identification of seasonal peaks in pathogen prevalence, as highlighted in our study, serves as a crucial guide for timing public health interventions to effectively reduce the transmission of these infections. By integrating these insights into the development of both individual patient care strategies and broader public health policies, our study is poised to substantially enhance the quality of healthcare services and the effectiveness of infectious disease containment efforts across the region. Through the strategic application of our findings, especially in the realms of immunization and infection prevention, we are charting a course toward a more resilient healthcare system capable of responding adeptly to the challenges posed by respiratory infections in children.

## 5 Conclusions

In conclusion, our extensive retrospective analysis has yielded important insights into the prevalence of respiratory pathogens in children within the southern Sichuan region. The discovery of a 52.8% pathogen detection rate highlights the critical role of precise diagnosis in the effective management of RTIs. Although gender did not significantly influence the overall presence of pathogens when adjusted for age, the gender-specific prevalence of certain pathogens calls for further investigation into the immunological and behavioral factors involved. Our study identifies RSV and IBV as the predominant viruses affecting this demographic, with age and seasonality influencing infection rates, offering valuable information for clinical and public health policy. The unusual prevalence of IBV and MP during spring and summer challenges the conventional understanding of these pathogens as predominantly winter conditions, suggesting that pathogen activity is influenced by more than just the cold season. Additionally, our data on the role of co-infections in increasing the risk of pneumonia emphasize the importance of careful clinical management, especially concerning MP, to prevent the worsening of the disease in children. By highlighting the importance of viruses and atypical pathogens in leading to pneumonia, this study informs healthcare strategies and underlines the necessity of public health measures to control the spread of respiratory infections. Future efforts should focus on enhancing diagnostic precision, optimizing treatment protocols, and bolstering prevention methods that are attuned to the specific epidemiological patterns of RTIs in southern Sichuan.

## Data Availability

The raw data supporting the conclusions of this article will be made available by the authors, without undue reservation.
